# Endothelial TLR4 at the Crossroads of Vascular Senescence and Targeted Senotherapeutics

**DOI:** 10.1111/acel.70757

**Published:** 2026-10-06

**Authors:** Hyo‐Jin Kim, Jeong‐Hyung Lee, Cheol Hwangbo

**Affiliations:** ^1^ Division of Life Science, College of Natural Sciences Gyeongsang National University Jinju Republic of Korea; ^2^ Plant Molecular Biology and Biotechnology Research Center (PMBBRC) and Research Institute of Life Sciences Gyeongsang National University Jinju Republic of Korea; ^3^ Department of Biochemistry (BK21 Four), College of Natural Sciences Kangwon National University Chuncheon Republic of Korea; ^4^ Division of Applied Life Science (BK21 Four), Research Institute of Life Sciences Gyeongsang National University Jinju Republic of Korea

**Keywords:** endothelial cells, senomorphic therapy, targeted drug delivery, TLR4, vascular aging

## Abstract

Traditionally viewed as a classic sentinel of innate immunity, Toll‐like receptor 4 (TLR4) has emerged as a crucial pathological driver within the vascular endothelium, with its identity dictated by cell‐type specificity, microenvironmental context, and fluctuating expression levels. This review critically examines the dual functional nature of endothelial TLR4, delineating how it transitions into a primary driver of vascular aging and organ‐specific vascular pathologies. The profound spatial, temporal, and cellular heterogeneities of TLR4 signaling expose the fundamental inadequacies of conventional systemic pan‐TLR4 blockade, which has repeatedly failed in clinical trials due to severe immunosuppression and metabolic disruptions. To bypass these limitations, we propose a precision senotherapeutic framework utilizing advanced drug delivery systems—such as Vascular cell adhesion molecule‐1 (VCAM‐1) or E‐selectin‐targeted nanoparticles and genetically engineered plant‐derived exosomes—to compartmentalize TLR4 inhibition within pathologically altered endothelial sub‐populations. By shifting the clinical paradigm from indiscriminate receptor suppression to spatiotemporal fine‐tuning, this conceptual framework provides a rational blueprint for next‐generation anti‐aging therapies aimed at restoring vascular homeostasis throughout the aging process.

## Graphical abstract

While Toll‐like receptor 4 (TLR4) mediates essential innate immune responses, its stress‐induced hyperactivation drives vascular endothelial senescence. The subsequent release of the senescence‐associated secretory phenotype (SASP) accelerates tissue aging and exacerbates various age‐related diseases. Because systemic TLR4 blockade compromises vital immune functions, we propose precision anti‐aging interventions—utilizing targeted delivery platforms such as nanoparticles and exosomes—to specifically neutralize TLR4 within senescent endothelial cells. These strategies overcome the limitations of conventional therapies, offering a conceptual blueprint for next‐generation vascular senotherapeutics.

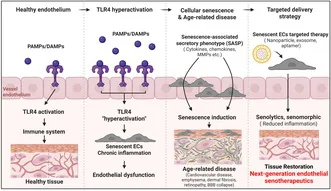

## 1 Introduction

The human vascular system functions as a dynamic network designed to maintain systemic tissue homeostasis by regulating nutrient delivery, oxygenation, and metabolic waste clearance (Hennigs et al. 2021). Far beyond serving as passive conduits, endothelial cells (ECs) form an active, semi‐permeable metabolic and endocrine monolayer capable of processing complex biochemical cues to modulate vascular tone, leukocyte trafficking, and fluid permeability (Kruger‐Genge et al. 2019).

Driven by telomere attrition, oxidative stress, and DNA damage, cellular senescence is defined by an irreversible cell cycle arrest and the robust secretion of a pro‐inflammatory cocktail known as the senescence‐associated secretory phenotype (SASP), comprising cytokines, chemokines, and matrix‐degrading enzymes (Khavinson et al. 2022). Phenotypically, these senescent cells exhibit a flattened morphology, upregulated expression of cell cycle inhibitors such as p53 and p16^INK4a^, and significantly increased senescence‐associated beta‐galactosidase (SA‐β gal) activity (Ajoolabady et al. 2025). SASPs can have negative effects on vascular function, such as accelerating the senescence of surrounding cells, inducing inflammatory responses, disrupting the blood‐vascular barrier, and promoting blood clot formation (Liberale et al. 2020). Endothelial senescence serves as the critical incipient stage for a wide spectrum of chronic diseases, precipitated by oxidative stress, metabolic disturbances, chronic inflammation, and chronological aging (Ting et al. 2021). Endothelial senescence profoundly exacerbates cardiovascular pathologies; mechanically, it diminishes nitric oxide (NO) bioavailability, which invariably leads to vasoconstriction, arterial stiffening, and subsequent hypertension (Seals et al. 2011). Furthermore, the disruption of endothelial junctional integrity facilitates the pathological infiltration of circulating macromolecules. Concurrently, senescent cells actively secrete SASP factors, which recruit immune cells to the vascular wall and induce bystander senescence in neighboring healthy cells, thereby severely compromising overall vascular function (Han and Kim 2023). This endothelial senescence triggers lipid accumulation and arterial hardening during atherosclerosis and diminishes NO bioavailability, leading to impaired vasorelaxation and subsequent hypertension (Scioli et al. 2020). Deciphering the upstream molecular triggers that govern this transition from homeostatic endothelium to a self‐destructive barrier is therefore paramount for developing effective vascular interventions.

Toll‐like receptor 4 (TLR4) is a type of pattern recognition receptor (PRR), well known as a crucial sensor of the innate immune system, binding to external pathogens or internally derived substances to induce an immune response (Janeway Jr. 1989). TLR4 stands at the nexus of this phenotypic shift, functioning as both a fundamental surveillance system and a pathological driver of vascular inflammation (Murad 2014). Under physiological conditions, endothelial TLR4 acts as a sentinel that detects invading pathogens and coordinates initial tissue repair, angiogenesis, and vascular wall reconstruction (Akira et al. 2006; Bezhaeva et al. 2022). However, under persistent metabolic stress—such as prolonged exposure to hyperglycemia or oxidized low‐density lipoprotein (oxLDL)—this protective architecture shifts into a maladaptive state. Chronic TLR4 hyperactivation fuels a robust pro‐inflammatory cascade, accelerating oxidative stress and uncoupling endothelial nitric oxide synthase (eNOS), which ultimately culminates in severe endothelial senescence and dysfunction (Kumar et al. 2025; Stierschneider and Wiesner 2023). This review analyzes the site‐specific endothelial senescence paradox induced by TLR4, which is distinct from its general immunological function. Furthermore, it presents the potential of TLR4 as an anti‐aging therapeutic target utilizing next‐generation nano‐convergence and delivery technology.

## 2 TLR4 Signaling Pathway

### 2.1 Endothelial TLR4 in Driving Cellular Senescence

Within the vascular tree, TLR4 exhibits its highest basal expression in ECs, positioning the endothelium as a primary immune‐regulatory hub that bridges innate intravascular signaling with systemic inflammation (Hijiya et al. 2002; Stierschneider and Wiesner 2023). Under homeostatic conditions, low‐level TLR4 signaling supports selective permeability, leukocyte rolling for protective immunity, and basic cellular defense functions (Kaisho and Akira 2006; Lee and Seong 2009). In addition, TLR4 activated by Damage‐associated molecular patterns (DAMPs) generated by cell damage induces the expression of Vascular endothelial growth factor (VEGF), an angiogenesis‐inducing factor, thereby stimulating endothelial cell migration and tube formation for tissue damage repair (Riddell et al. 2012). The basic expression level of TLR4 in endothelial cells is essential for the oxidative stress defense mechanism induced by VEGFR2, as well as for maintaining homeostasis and protection of lung tissues (Takyar et al. 2016). However, hyperactivation of this pathway causes the vascular defense system to become self‐destructive, orchestrating a progressive slide into immunoaging, localized cytokine storms, and barrier breakdown (Goulopoulou et al. 2016; S. J. Kim et al. 2019).

When persistently stimulated by circulating DAMPs or oscillatory shear stress (OSS), endothelial TLR4 triggers a self‐reinforcing vicious cycle that drives eNOS dysfunction, oxidative stress, and a transition into replicative cellular senescence. Mechanistically, TLR4 signaling activates the NF‐κB pathway via MyD88/TRIF adapters while simultaneously inducing the assembly of the NADPH oxidase 2 (NOX2) complex, resulting in massive superoxide production (F. Wu et al. 2014). This oxidative surge keeps eNOS inactive, consequently drastically reducing local NO bioavailability and generating highly toxic nitric oxide (Karbach et al. 2014; Wang et al. 2019). The accumulation of intracellular oxidative stress induces double‐stranded DNA damage, activating the classic DNA damage response (DDR). Phosphorylation of p53 by ATM kinase subsequently induces the transcription of cyclin‐dependent kinase (CDK) inhibitors, which are cell cycle regulatory proteins, specifically p21^CIP1/WAF1^ and p16^INK4a^ (Liu, Chu, and Wu 2021). This increased expression of these CDK inhibitors (CDKIs) drives endothelial cells into an irreversible G1‐phase cell cycle arrest, which is a characteristic of replicative senescence.

Senescent ECs exhibit an up‐regulated surface expression of TLR4 and amplified basal NF‐κB signaling, creating a “hyper‐inflammatory” phenotype that displays a ninefold upregulation of ICAM‐1 upon secondary LPS stimulation compared to healthy controls (Abdelgawad et al. 2023; Budamagunta et al. 2021; Suzuki et al. 2022). Once senescent, these endothelial cells persistently secrete a broad spectrum of SASP factors—including IL‐6, IL‐1α/β, IL‐8, TNF‐α, MCP‐1, ICAM‐1, VCAM‐1, and MMPs—fueled by sustained NF‐κB and p38 MAPK activation (Suzuki et al. 2019; Suzuki et al. 2022; H. Zhou et al. 2023). The secreted SASP factors not only induce bystander senescence in neighboring healthy cells but also feedback to further upregulate TLR4 expression on the senescent cells themselves, establishing a permanent, self‐amplifying pathological loop (Janaszak‐Jasiecka et al. 2023; Wang et al. 2019) (Figure 1).

**FIGURE 1:**
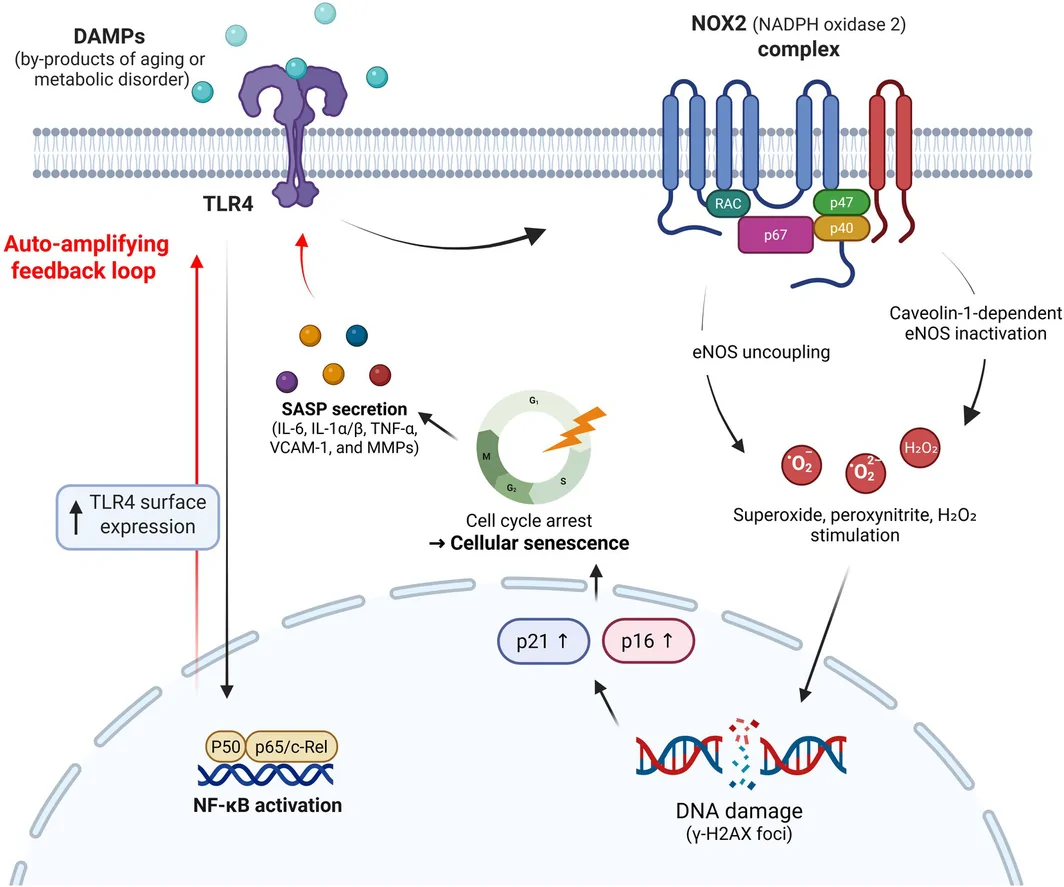
Molecular mechanisms of endothelial TLR4‐mediated senescence and the SASP self‐amplifying loop. Chronic microenvironmental exposure to DAMPs, PAMPs, or oscillatory shear stress (OSS) drives persistent TLR4 activation within the endothelial monolayer. Downstream signaling through MyD88/TRIF adaptors triggers NF‐κB nuclear translocation to initiate pro‐inflammatory transcription, while simultaneously assembling the NADPH oxidase 2 (NOX2) complex to induce massive reactive oxygen species (ROS) accumulation. Sustained oxidative stress precipitates DNA double‐strand breaks (γ‐H2AX foci), activating the ATM‐p53 checkpoint pathway. Subsequent upregulation of cyclin‐dependent kinase (CDK) inhibitors p21^CIP1/WAF1^ and p16^INK4a^ maintains the retinoblastoma (Rb) protein in a hypophosphorylated state, enforcing permanent G1‐phase replicative senescence. Senescent endothelial cells develop a hyper‐inflammatory phenotype characterized by baseline TLR4 upregulation and the robust secretion of senescence‐associated secretory phenotype (SASP) factors (e.g., IL‐6, IL‐1β, TNF‐α), which propagate paracrine bystander senescence to adjacent healthy cells and perpetuate a self‐reinforcing pathological loop. Created with http://biorender.com/.

Considering the above, TLR4 demonstrates value as a therapeutic target for diseases resulting from endothelial senescence. Contemporary senotherapeutic strategies primarily encompass two distinct modalities: senolytics, which selectively induce apoptosis in senescent cells, and senomorphics. Senomorphic approaches aim to reprogram senescent cells by inhibiting key secretory signaling pathways (e.g., NF‐κB, mTOR), thereby neutralizing the toxic SASP cocktail that otherwise drives bystander senescence in adjacent tissues (Imawari and Nakanishi 2024; Noh et al. 2024). Therefore, analyzing the function and mechanism of TLR4 in endothelial cells of each tissue is crucial for determining how to utilize it as a target for comprehensive senotherapeutic interventions.

### 2.2 TLR4 as an Immune System Regulator

The vascular endothelium encounters a continuous influx of both exogenous pathogens and endogenous stress signals, both of which converge on TLR4 to dictate whether the resulting cascade is a protective, acute defense mechanism or a chronic, maladaptive condition (Newton and Dixit 2012). While Pathogen‐associated molecular patterns (PAMPs)—such as lipopolysaccharide (LPS), flagellin, and viral RNA—are exogenous microbial motifs that orchestrate acute defensive immunity, DAMPs represent endogenous danger signals released exclusively during cellular injury, stress, or necrotic cell death; these include nuclear HMGB1, mitochondrial DNA, and uric acid crystals, which function primarily to initiate tissue repair and the clearance of damaged cellular debris (Tang et al. 2012). During acute infections, PAMP‐mediated activation—most notably by LPS—triggers a highly coordinated immune response. At the plasma membrane, it recruits the adaptor protein MyD88, leading to the rapid activation of NF‐κB and the robust secretion of pro‐inflammatory cytokines such as TNF‐α and IL‐6 (Kawai et al. 2001). Subsequently, the receptor complex is internalized into endosomes, shifting to a TRIF‐dependent pathway that activates IRF3 to induce Type I interferons (Kagan et al. 2008). This coordinated dual‐signaling architecture effectively bridges innate and adaptive immunity to clear the pathogen (Kawai and Akira 2010). Conversely, chronic exposure to endogenous DAMPs—such as oxLDL, HMGB1, and saturated fatty acids—initiates a low‐grade, persistent pathological state termed “sterile inflammation” (Andersson and Tracey 2011; Rocha et al. 2016). DAMP‐mediated activation frequently bypasses traditional co‐receptors or cross‐talks with alternative receptors like RAGE, biassing the receptor response toward a chronic MyD88/NF‐κB axis without the acute intensity of pathogen clearance (Chun and Seong 2010; Schmidt et al. 2001). RAGE, similar to TLR4, serves as a promiscuous receptor for intracellular DAMPs (e.g., HMGB1, S100 proteins) and advanced glycation end products (AGEs), amplifying signals of vascular aging and chronic inflammation (Schmidt et al. 1999). Consequently, isolating these ligand‐specific contexts is critical for designing targeted therapies that selectively disrupt sterile inflammation while preserving acute host defense.

## 3 The Correlation of Endothelial TLR4 and Disease

Endothelial senescence profoundly alters the expression profile and basal activity of TLR4, positioning this receptor as a pivotal mediator in the progression of age‐related vascular pathologies. During chronological aging, diverse DAMPs—including cell‐free DNA, mitochondrial debris, and AGEs—systemically accumulate (Piccinini and Midwood 2010). These circulating DAMPs persistently engage endothelial TLR4 to activate NF‐κB, triggering the robust secretion of pro‐inflammatory cytokines (e.g., TNF‐α, IL‐1β) and interferons. This inflammatory milieu consequently establishes a vicious cycle by further upregulating endothelial TLR4 expression (Calvo‐Rodriguez et al. 2017; H. J. Kim et al. 2023). In human aortic smooth muscle cells (HASMCs), it was confirmed that LPS‐induced TLR4 activation increases TLR4 mRNA transcription and stability via NF‐κB, representing a post‐transcriptional regulatory mechanism of the TLR4 gene (Lin et al. 2006). Although changes in TLR4 expression with aging have been observed in animal models and endothelial cells, the precise regulatory mechanisms underlying this expression remain poorly understood (S. J. Kim et al. 2019; Liu, Chu, and Wu 2021). Therefore, understanding the role and function of TLR4 in diseases caused by endothelial aging is crucial for considering TLR4 as an anti‐aging therapeutic target.

### 3.1 Endothelial TLR4 and Age‐Related Disease

Fluctuations in vascular endothelial TLR4 expression and activity determine tissue‐specific inflammatory vulnerability, positioning the receptor as a chronic pathogenic driver of age‐associated cardiovascular disease. In aging mouse models, chronological aging directly correlates with a significant upregulation of TLR4 levels within the heart and aorta, accelerating localized vascular decline (Liu, Chu, and Wu 2021). Vascular inflammatory sensitivity is highly site‐specific: microvascular ECs express significantly higher basal CD14, a crucial co‐receptor for TLR4, than macrovascular ECs, triggering an amplified IL‐6 cascade that drives macrophage MMP‐1 production and subsequent atherosclerotic plaque destabilization (Lu et al. 2012). Endogenous DAMPs generated under metabolic or mechanical stress converge on the TLR4 signaling pathway, perpetuating endothelial damage. In angiolipomas, extracellular galectin‐3 oligomerizes upon binding to surface β‐galactoside residues, forming a physical ‘gal‐glycan lattice’ on the endothelial membrane that hyperactivates TLR4, driving abnormal microvascular proliferation and microthrombosis (Arciniegas et al. 2024). During diabetic vascular disease, advanced glycation end‐products upregulate both TLR4 and its co‐ligand HMGB1, establishing a feedforward loop where RAGE activation continuously amplifies MyD88/TIRAP/NF‐κB‐mediated mitochondrial dysfunction (Ramya et al. 2021). In large arteries, oxLDL promotes CD36–TLR4 receptor crosstalk and accelerates plaque progression, while oxidized phospholipids (oxPAPC) and free fatty acids similarly trigger persistent endothelial cytokine release (Menghini et al. 2014; Su et al. 2024).

According to studies using cell type‐specific genetic engineering in emphysema models, endothelial TLR4 maintains lung structural integrity by inhibiting the senescence gene p16^INK4a^ through HDAC2‐mediated histone H4 deacetylation. A deficiency in endothelial TLR4 precipitates endothelial senescence and p16^INK4a^‐driven alveolar dilation (S. J. Kim et al. 2019). In acute respiratory distress syndrome, extracellular eNAMPT binds to endothelial TLR4, downregulating the junctional proteins Dock1 and Elmo1 to drive severe pulmonary edema and vascular leakage (Song et al. 2022). Collectively, these findings suggest that the expression and activity of TLR4 in lung ECs are important for the development of age‐related diseases.

Senescent ECs extend their pathogenic reach to distal tissues through extracellular vesicle‐mediated paracrine communication. For instance, senescent ECs release exosomes containing limited amounts of miR‐326‐3p due to a selective cargo sorting network regulated by specific RNA‐binding proteins (RBPs) and exo‐motifs (Yang et al. 2022). When absorbed by skin fibroblasts, this restricted miRNA delivery fails to properly regulate fibroblast homeostasis, modulating downstream apoptotic pathways and accelerating dermal aging. Within the macrovasculature, endothelial TLR4 serves as a critical mediator of angiotensin II (Ang II)‐induced vascular remodeling. TLR4 signaling promotes NADPH oxidase activation, generating an oxidative environment that sustains hypertension‐related vascular damage independently of systemic blood pressure elevation (Hernanz et al. 2015). In the microvasculature, tissue‐specific genetic deletion has revealed that endothelial TLR4 acts as the primary driver of diabetic retinopathy. Endothelial‐specific TLR4 deletion confers profound protection against retinal vascular permeability, neuronal damage, and capillary degeneration; conversely, deleting TLR4 in adjacent Müller cells merely reduces localized inflammatory cytokines without halting structural microvascular breakdown, confirming that endothelial TLR4 directly dictates blood‐retinal barrier integrity (Seidel et al. 2021).

Hyperactivation of endothelial TLR4 by exogenous PAMPs or endogenous DAMPs disrupts crucial physiological barriers and plaque stability across various vascular beds. For example, acute PAMP‐mediated TLR4 activation in brain capillary endothelial cells induces the rapid internalization of the tight junction protein claudin‐5 into endocytic compartments, causing blood–brain barrier breakdown and catastrophic vascular collapse—a phenomenon entirely absent in endothelial‐specific TLR4 knockout (Tlr4^ECKO^) mice (Seegren et al. 2026). In systemic metabolic conditions, endothelial TLR4 activation by PAMPs directly suppresses eNOS expression, leading to localized ischemia and microvascular perfusion failures that exacerbate neonatal necrotizing enterocolitis (Yazji et al. 2013) (Table 1).

**TABLE 1:** Pathogenic roles of vascular endothelial TLR4 in tissue‐specific diseases.

Site of disease	Role of TLR4	Key cell type	Major mechanism	References
Vascular disease	Chronic pathogenic driver	Heart and aorta endothelial cells	Basal TLR4 expressions increase chronologically, amplifying age‐dependent inflammatory cascades	Liu, Chu, and Wu (2021)
	Determinant of inflammatory sensitivity	Microvascular endothelial cells	High basal CD14 (TLR4 co‐receptor) triggers the IL‐6 cascade	Lu et al. (2012)
	Hyperactivation target via lattice formation	Angiolipomas	Extracellular galectin‐3 binds to β‐galactoside, forming a ‘gal‐glycan lattice’	Arciniegas et al. (2024)
	Feedforward loop mediator	Diabetic vessel	AGEs upregulate TLR4 and HMGB1, activating RAGE	Ramya et al. (2021)
	Crosstalk receptor & stress sensor	Large arteries	oxLDL promotes CD36–TLR4 cross‐talk; oxPAPC and FFAs act as DAMPs	Menghini et al. (2014); Su et al. (2024)
Lung	Homeostatic protector	Lung endothelial cells versus epithelial cells	Endothelial TLR4 maintains structural integrity by suppressing p16^INK4a^ via HDAC2‐mediated H4 deacetylation	S. J. Kim et al. (2019)
	Endothelial barrier disrupter	Lung (acute respiratory distress syndrome)	Extracellular eNAMPT binds to endothelial TLR4, downregulating junctional proteins Dock1 and Elmo1	Song et al. (2022)
Skin	Paracrine aging modulator	Senescent endothelial cells → dermal fibrosis	Altered exosomal miR‐326‐3p sorting from senescent ECs leads to dysregulated fibroblast apoptosis and senescence	Yang et al. (2022)
Retina	Primary driver of microvascular injury	Retinal endothelial cells versus Müller cells	Endothelial TLR4 drives capillary degeneration and permeability; Müller cell TLR4 contributes only to ancillary cytokine release	Seidel et al. (2021)
Brain	Blood–brain barrier (BBB) destabilizer	Brain capillary endothelial cells	Acute PAMP‐mediated activation induces rapid internalization of tight junction protein claudin‐5 into endocytic compartments	Seegren et al. (2026)
Intestines	Microvascular perfusion inhibitor	Intestinal microvasculature	PAMP‐mediated activation directly suppresses eNOS expression	Yazji et al. (2013)

## 4 The Context‐Dependent Paradox of TLR4 in Vascular Aging

Accumulating evidence indicates that TLR4 lacks a monolithic directionality during vascular aging, instead presenting a highly nuanced, context‐dependent duality (Murad 2014). This functional friction challenges the conventional view of TLR4 as a simple pro‐inflammatory marker and exposes why unguided therapeutic interventions fail (Crammond et al. 2026). These variations reflect tissue‐specific specializations, distinctively highlighted when comparing cerebral and peripheral endothelial cells. While the cerebral endothelium restricts paracellular transport via sophisticated tight junctions and utilizes selective channels for glucose and iron transport, the peripheral endothelium maintains flexible permeability for macromolecules, hormones, and cells, rapidly modulating adhesion molecules for immune cell trafficking (Aird 2007; D. Wu et al. 2023). These functional differences support the hypothesis that the correlation between endothelial senescence and TLR4 activation may be tissue specific. Microvascular ECs exhibit a tenfold higher baseline NF‐κB sensitivity and elevated CD14 expression upon TLR4 stimulation compared to macrovascular ECs, shifting the pathological outcome toward plaque rupture via localized MMP‐1 secretion (Lu et al. 2012) (Figure 2A). This spatial divergence is further complicated by a distinct cell‐specific paradox within the same organ microenvironment. In diabetic retinopathy, endothelial‐specific TLR4 deletion halts microvascular breakdown, whereas Müller cell‐specific knockout fails to protect the blood‐retinal barrier, despite both cell types existing in identical hyperglycemic conditions (Seidel et al. 2021) (Figure 2B). A reciprocal cell‐specific paradox occurs in the lungs: systemic TLR4 deletion triggers spontaneous emphysema due to the loss of p16^INK4a^ suppression, and structural recovery is achieved exclusively by restoring TLR4 expression in the lung endothelium, not the alveolar epithelium (S. J. Kim et al. 2019) (Figure 2C). These findings demonstrate that endothelial TLR4 governs crucial homeostatic blueprints that cannot be replicated by adjacent parenchymal cells.

**FIGURE 2:**
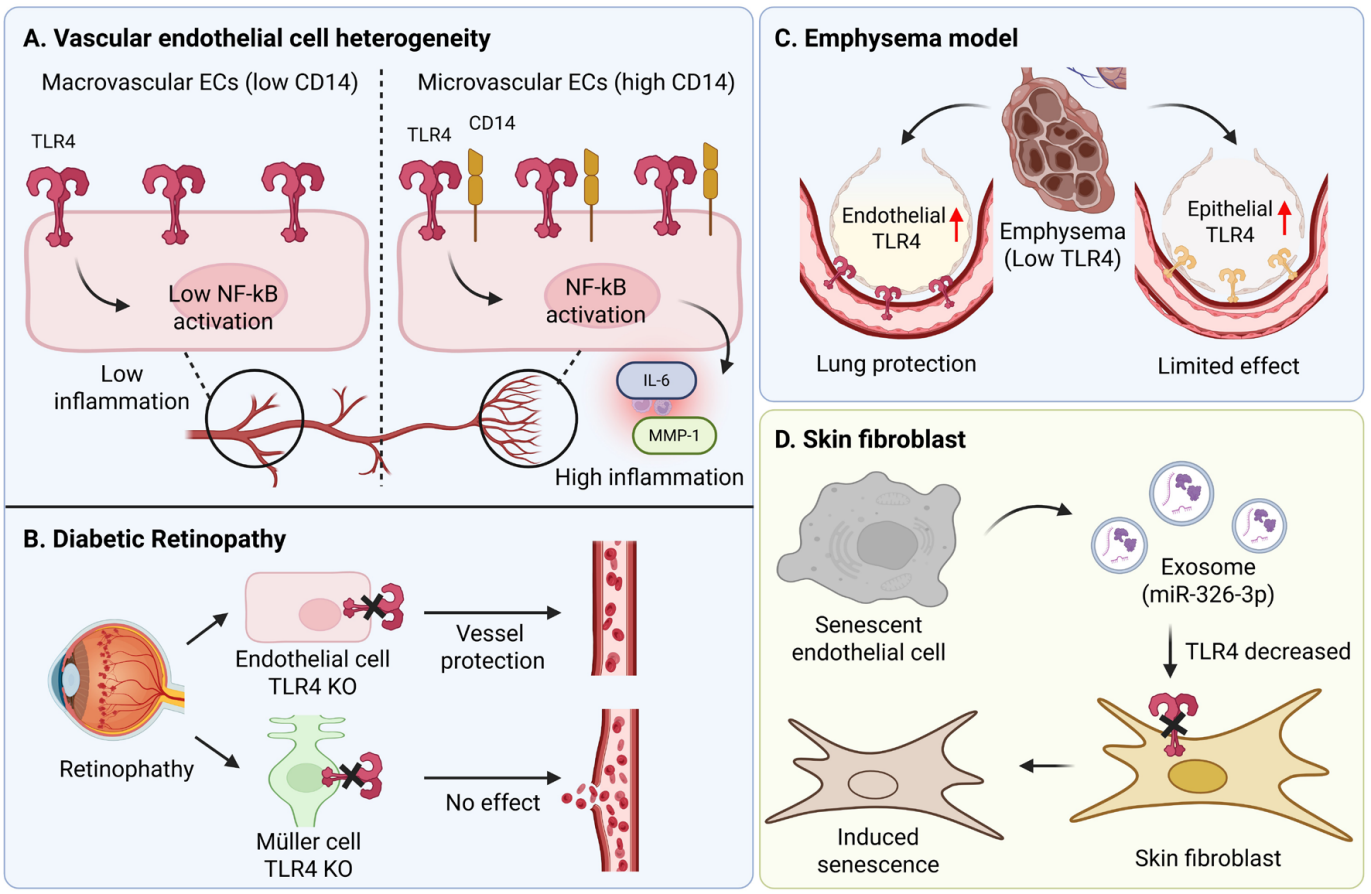
Cellular, spatial, and expression paradoxes of TLR4 signaling in vascular aging. (A) Spatial divergence between microvascular and macrovascular endothelial cells; elevated basal CD14 expression in microvessels confers a tenfold higher sensitivity to NF‐κB activation and subsequent plaque destabilization via IL‐6 and MMP‐1. (B) Cell‐type specificity in diabetic retinopathy; endothelial‐specific TLR4 deletion rescues blood‐retinal barrier integrity, whereas Müller cell‐specific knockout fails to prevent capillary regression under identical hyperglycemic stress. (C) Functional dichotomy in emphysema models, where endothelial TLR4 maintains alveolar structural integrity via HDAC2‐mediated epigenetic mechanisms, while epithelial TLR4 affords only limited tissue protection. (D) The expression paradox of intercellular senescence. Endothelial senescence upregulates baseline TLR4 expression, driving local vascular dysfunction. Conversely, senescent endothelial cells restrict the release of exosomal miR‐326‐3p to selectively downregulate TLR4 in adjacent dermal fibroblasts. The resulting TLR4 deficiency impairs basal cytoprotection and paradoxically accelerates fibroblast aging. Created with http://biorender.com/.

Superimposed on these cellular discrepancies is a striking expression paradox that governs intercellular senescence. In senescent ECs, chronological aging upregulates TLR4 expression, driving chronic vascular decline through SASP over‐secretion (Liu, Chu, and Wu 2021). Paradoxically, these same senescent ECs package and limit the release of exosomal miR‐326‐3p, which downregulates TLR4 within adjacent dermal fibroblasts (Yang et al. 2022). Within these fibroblasts, the resulting TLR4 deficiency impairs basal survival signaling and accelerates cellular senescence (Figure 2D). Thus, the exact same aging phenotype is driven by an excess of TLR4 in the endothelium and a deficit of TLR4 in neighboring fibroblasts. Ultimately, endothelial TLR4 possesses a dual identity, functioning simultaneously as a localized tissue damage promoter and an indispensable anti‐aging regulator. Its biological outcome is determined strictly by its vascular topography, cell type, and baseline expression thresholds, indicating that simple systemic inhibition or activation strategies are fundamentally flawed (Figure 2).

## 5 TLR4 Targeting Therapy

### 5.1 Therapeutic Effect by Systemic TLR4 Regulation

In therapeutic strategies targeting TLR4, the clinical paradigm must shift from a broad inhibition of acute pathogen defense toward the selective suppression of TLR4 upregulation driven by chronic sterile inflammation. Preserving the receptor's capacity to mount vital immune responses against acute infection, while specifically mitigating the persistent DAMP‐mediated signaling cascade, represents a critical prerequisite for effective anti‐aging and anti‐inflammatory interventions. In the cardiovascular system, neutralizing anti‐TLR4 antibodies in spontaneously hypertensive rats successfully reduces systemic blood pressure and restores acetylcholine‐dependent vasorelaxation, while the small‐molecule inhibitor CLI‐095 limits Ang II‐induced NADPH oxidase activity and vascular smooth muscle proliferation (De Batista et al. 2014). A recent preprint suggests that CLI‐095 treatment in ApoE^−/−^ mice suppresses nilotinib‐exacerbated endothelial inflammation and atherosclerotic plaque progression (Qu et al. 2019). Beyond direct vascular protection, systemic TLR4 blockade with TAK‐242 reverses chronic high‐fat diet‐induced insulin resistance in rodent models and attenuates hepatic fibrosis by suppressing the TLR4/MyD88/NF‐κB axis along the liver‐gut axis (Zhang et al. 2015).

Human cohort analyses further corroborate that genetically blunted TLR4 signaling confers protection against age‐related vascular decline. A study of a human cohort consisting of 2679 patients with coronary artery disease (CAD) aged 50–80 years demonstrated that while markers of vascular aging typically progress chronologically, carriers of the loss‐of‐function TLR4 variant rs4986790 (896A/G) exhibit a significantly attenuated trajectory of systolic blood pressure elevation over time (Schneider et al. 2015). Additionally, a 5‐year follow‐up study by an Italian research team on 810 subjects showed that compared to subjects with normal TLR4, 55 subjects with the Asp299Gly TLR4 allele had lower expression of specific inflammatory cytokines and acute‐phase reactants, such as interleukin‐6 and fibrinogen, and a reduced risk of atherosclerosis (Kiechl et al. 2002). Cohort studies on the very elderly population also confirmed that genetic profiles inhibiting TLR4 activity tend to contribute to longevity through inflammation control (Balistreri et al. 2009). These diverse preclinical successes firmly validate the TLR4 signaling axis as a highly actionable therapeutic target.

### 5.2 Side Effects Caused by Systemic TLR4 Regulation

Despite robust preclinical validation, transferring systemic pan‐TLR4 inhibition into clinical practice has failed due to the receptor's pleiotropic and homeostatic roles across multiple organ systems. Large‐scale Phase III clinical trials evaluating the competitive MD‐2/TLR4 antagonist eritoran and the intracellular TIR‐domain blocker TAK‐242 failed to reduce 28‐day mortality in severe sepsis patients, forcing early trial terminations (Opal et al. 2013; Rice et al. 2010). Complete abrogation of systemic TLR4 signaling dismantles the baseline innate immune defense required for pathogen clearance; this is illustrated by TLR4‐deficient mice, which suffer from defective neutrophil recruitment and blunted TNF‐α and IL‐12 production, resulting in fatal gram‐negative pneumonia. Furthermore, systemic TLR4 ablation induces severe, age‐dependent metabolic and mechanical side effects. While young TLR4 knockout (KO) mice resist diet‐induced obesity, aged TLR4 KO mice develop spontaneous obesity and visceral adiposity, driven by an imbalanced Th1/Th2/Th17 immune profile and a paradoxical macrophage polarization shift from anti‐inflammatory M2 to pro‐inflammatory M1 phenotypes (Z. Y. Zhou et al. 2022). Mechanical load models reveal a similar functional split: while systemic TLR4 deficiency protects against high‐fat diet‐induced cardiac hypertrophy, it paradoxically worsens cardiac performance under mechanical pressure overload conditions, resulting in reduced ejection fraction and progressive myocardial fibrosis (Tian et al. 2023). In oncology, systemic TLR4 blockade poses a parallel double‐edged sword: while it reduces chronic tumor‐permissive inflammation, it simultaneously disrupts dendritic cell maturation and cytotoxic T‐cell priming, accelerating tumor growth in surveillance models (Awasthi 2014). These broad‐spectrum liabilities demonstrate that untargeted systemic inhibition is an unviable clinical strategy, underscoring the urgent need for cell‐type‐specific or context‐tailored interventions.

### 5.3 Targeting Endothelial TLR4 via Genetic Ablation

Compartmentalizing TLR4 inhibition specifically within the vascular endothelium circumvents the systemic toxicities of pan‐TLR4 blockade while yielding profound therapeutic benefits. In experimental meningitis models, endothelial‐specific TLR4 knockout mice achieve a near‐complete blockade of intravascular IL‐6, CXCL10, and ICAM‐1, demonstrating that the endothelium acts as the primary transducer of systemic inflammatory injury rather than a passive responder (Seegren et al. 2026). This dominant paracrine influence is mirrored in diabetic retinopathy, where endothelial‐specific TLR4 deletion selectively rescues the retinal microvasculature from regression and neurodegeneration, an effect unachievable by targeting adjacent Müller cells (Seidel et al. 2021). Similarly, endothelial‐specific TLR4 restoration in emphysema models achieves complete phenotypic recovery and lung integrity rescue by normalizing downstream p16^INK4a^ aging pathways, highlighting the superior efficacy of cell‐targeted modulation (S. J. Kim et al. 2019) (Table 2A).

**TABLE 2:** Endothelial TLR4‐targeted therapeutic strategies.

Category	Agent/Strategy	Molecular target	Key functional effect	Disease context
A. Therapeutic strategies through genetic ablation				
TLR4 knockdown	CRISPR‐Cas9	Endothelial‐specific TLR4 deletion	Blocks primary vascular cytokine transduction and leukocyte recruitment	Neonatal meningitis defense
TLR4 knockdown	Transgenic mouse	Endothelial TLR4 overexpression	Rescues HDAC2 activity, repressing p16^INK4a^ to halt lung tissue damage	Pulmonary emphysema rescue
TLR4 knockdown	Cre‐lox mouse	Retinal endothelial TLR4 deletion	Preserves capillary structural integrity and prevents neurodegeneration	Diabetic retinopathy protection
B. Senotherapeutic strategies				
Direct TLR4 antagonism	Eritoran	Endothelial membrane TLR4/MD‐2	Blunts acute LPS injury and disrupt chronological SASP propagation	LPS‐induced inflammation/Vascular aging
Direct TLR4 antagonism	Heparin	TLR4/MyD88 expression	Blocks NF‐κB nuclear translocation, exerting a direct senomorphic effect	Sterile inflammation/Age‐related decline
Downstream signaling	Sildenafil/Nitrites	eNOS‐NO‐Nitrite pathway	Restores NO bioavailability, bypassing MyD88‐mediated endothelial ischemia	Neonatal Necrotizing Enterocolitis
Downstream signaling	SAG (Smoothed agonist)	Hedgehog signaling pathway	Reactivates the HH pathway, halting TLR4‐mediated placental stem cell senescence	Placental vascular injury/Preeclampsia
Downstream signaling	Cerivastatin	HCAEC IL‐6 signaling axis	Selectively dampens TLR4‐activated IL‐6, IL‐8, and MCP‐1 release in coronary beds	Chronic Heart Failure/CAD
Upstream modulation	Ash2l inhibition	Epigenetic H3K4 trimethylation	Suppresses CD36‐TLR4 interaction, reducing lipid uptake and plaque size	Atherosclerosis (ApoE^−/−^)
Upstream modulation	RAGE inhibition	RAGE‐TLR4 cross‐talk	Downregulates absolute TLR4 expression, blocking both MyD88 and TRIF pathways	Diabetic micro‐ and macrovasculature
Ligand neutralization	Anti‐eNAMPT Antibody	Extracellular eNAMPT ligand	Preserves Dock1 and Elmo1 expressions, preventing acute pulmonary leakage	Acute lung injury/Pulmonary edema

### 5.4 Endothelial TLR4 as a Senotherapeutic Target

Pharmacological agents capable of fine‐tuning the endothelial TLR4 axis hold significant clinical promise when rebranded within a senomorphic framework. Clinically utilized heparin potently suppresses endothelial TLR4 and MyD88 expression in a dose‐dependent manner, blocking NF‐κB nuclear translocation and reducing SASP components (IL‐1β, IL‐6) to delay cellular senescence (Liu, Li, et al. 2021). The targeted antagonist eritoran similarly neutralizes LPS‐induced endothelial SASP (IL‐6, IL‐8) propagation (Menghini et al. 2014). Under mechanical stress, placental and uterine artery angiogenesis impaired by TLR4 hyperactivation can be fully rescued through the administration of the Smoothed agonist (SAG), which reactivates the Hedgehog pathway to halt endothelial senescence and reverse localized vascular defects (Zhong et al. 2022). Furthermore, sildenafil or nitrite supplementation effectively bypasses LPS‐mediated TLR4 injury by restoring baseline eNOS‐NO function and normalizing microcirculatory perfusion during neonatal necrotizing enterocolitis (Yazji et al. 2013). In coronary pathologies, cerivastatin directly suppresses TLR4‐activated IL‐6 secretion within human coronary artery endothelial cells (HCAECs), delaying the progression of chronic heart failure and coronary artery disease (Zeuke et al. 2002).

Advanced strategies are now moving beyond direct receptor blockade to target the upstream genes and ligands that govern endothelial TLR4 sensitivity. Epigenetic targeting of the Ash2l gene in ApoE^−/−^ mice selectively dampens oxLDL uptake and downregulates endothelial TLR4 assembly, stabilizing atherosclerotic plaque size and enhancing fibrous cap thickness (Su et al. 2024). In diabetic macro‐ and microvascular environments, silencing the RAGE gene successfully downregulates baseline TLR4 expression, simultaneously neutralizing both MyD88 and TRIF cascades to suppress chronic vascular complications (Ramya et al. 2021). Alternatively, ligand‐targeted neutralization using anti‐eNAMPT antibodies prevents the pathological downregulation of the vasoprotective molecules Dock1 and Elmo1, maintaining endothelial junctional integrity and protecting against severe pulmonary edema and acute lung injury (Song et al. 2022) (Table 2B).

## 6 Future Directions and Precision Senotherapeutic Strategies

Current clinical paradigms for mitigating age‐related vascular decline predominantly rely on broad lifestyle interventions, such as exercise and caloric restriction, or the off‐label use of pharmacological agents like metformin. While calorie restriction improves lipid metabolism and reduces triglyceride levels in aged mice, it fails to alleviate oxidative stress, thereby increasing age‐related inflammation (Teofilovic et al. 2022). Metformin, originally developed as an antidiabetic agent, exerts robust senomorphic effects by targeting key longevity signaling pathways (Chen et al. 2022). However, its net therapeutic benefit in endothelial senescence‐driven pathologies remains ambiguous due to concurrent anti‐angiogenic effects and systemic toxicities, such as lactic acidosis.

Endothelial TLR4 functions as a sophisticated molecular rheostat whose pathological or protective identity is determined entirely by spatial topography, cell‐type interaction, and expression boundaries. Attempting to neutralize its pathogenic effects through unguided systemic pan‐TLR4 blockade creates a major clinical dilemma: it dismantles crucial epigenetic anti‐aging pathways in the endothelium, disrupts survival signaling in adjacent fibroblasts, and compromises systemic immune surveillance, causing liabilities that consistently outweigh therapeutic benefits. This multi‐organ friction explains why systemic antagonists like TAK‐242 and eritoran have repeatedly failed in large‐scale clinical trials (Opal et al. 2013; Rice et al. 2010).

Consequently, the next generation of cardiovascular and anti‐aging therapies must transition away from absolute receptor inhibition toward precision platforms that confine TLR4 modulation strictly within pathologically altered endothelial sub‐populations, leaving homeostatic cell compartments untouched.

### 6.1 In Vivo Targeted Delivery Technologies as a Senotherapeutic Alternative

The restricted delivery of senescent‐type targets or TLR4 inhibitory targets to damaged or temporally aged vascular sites can be achieved using functionalized nanoplatforms that leverage proteins overexpressed on the surface of aged or activated ECs.

Confining senomorphic or TLR4‐silencing payloads strictly to damaged or chronologically aged vascular segments can be achieved using functionalized nanoplatforms that exploit surface proteins overexpressed on senescent ECs (Castro et al. 2024). Advanced nanostructured lipid carriers (NLCs) functionalized with anti‐VCAM‐1 antibodies selectively accumulate within inflamed endothelial segments, such as the aortic sinus and active atherosclerotic plaques, while completely bypassing healthy vessels (Distasio et al. 2021). Utilizing this specificity to deliver target cargoes—such as melatonin—successfully silences the downstream endothelial TLR4/NF‐κB cascade, suppressing NLRP3‐mediated pyroptosis and reversing acute lung injury in vivo (Jin et al. 2024). Parallel success has been achieved using maleimide‐modified nanoemulsions conjugated with anti‐VCAM‐1 fragments, providing a highly precise diagnostic and therapeutic vehicle for mapping and treating senescent vascular beds (Belcastro et al. 2021; Shi et al. 2024). For acute intravascular injuries or early‐stage interventions, E‐selectin serves as an alternative endothelial homing marker. Implementing the E‐selectin‐targeted multistep vector (ESTA‐MSV) system to deliver therapeutic microRNAs (e.g., miR‐146a and miR‐181b) directly to activated ECs achieves a robust downregulation of downstream vascular adhesion molecules and a significant reduction in atherosclerotic lesion size (Ma et al. 2016). To circumvent the heterogeneity of single‐adhesion‐molecule expression, dual‐targeting platforms have been developed. Conjugating both anti‐VCAM‐1 and anti‐E‐selectin antibodies to cationic lipid‐based SAINT‐O‐Somes ensures the highly selective delivery of targeted small interfering RNA (siRNA) into TNF‐α‐activated aortic and venous ECs, inducing complete gene silencing with zero detectable systemic cytotoxicity (Kowalski et al. 2013) (Figure 3).

**FIGURE 3:**
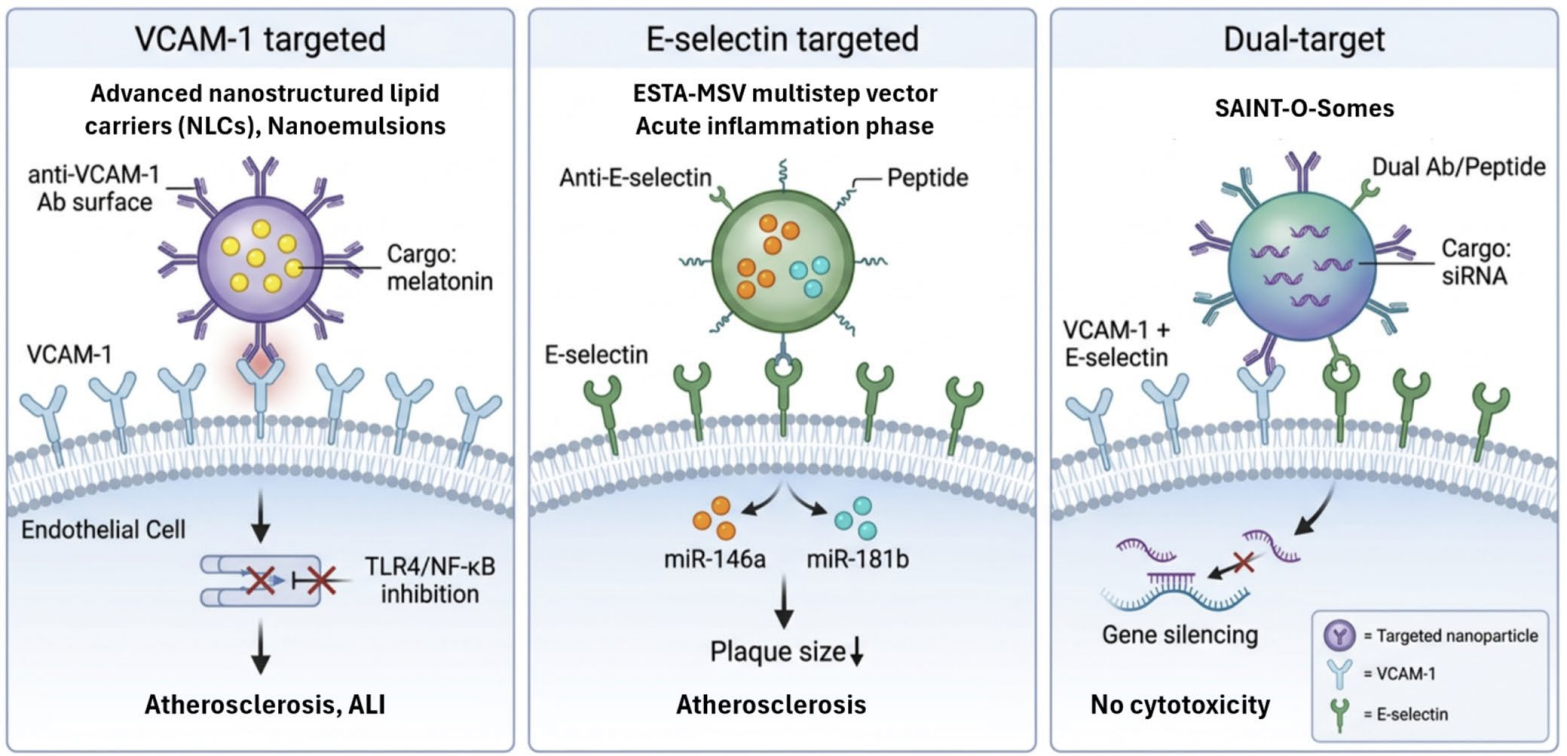
Nanoparticle‐based drug delivery systems functionalized for endothelial surface targeting. Schematic representation of targeted nanomedicine strategies utilizing adhesion molecules overexpressed on activated and senescent endothelial cells. (Left) VCAM‐1‐targeted nanostructured lipid carriers (NLCs) conjugated with anti‐VCAM‐1 antibodies selectively deliver therapeutic payloads (e.g., melatonin) to halt the TLR4/NF‐κB cascade, suppressing downstream NLRP3 pyroptosis and atherosclerotic lesion progression. (Middle) E‐selectin‐targeted multistep vectors (ESTA‐MSV) deliver regulatory microRNAs (e.g., miR‐146a/181b) during acute inflammatory phases to downregulate surface adhesion molecules and reduce plaque size. (Right) Dual‐targeting SAINT‐O‐Somes leverage multi‐ligand conjugation to simultaneously bind VCAM‐1 and E‐selectin, delivering targeted siRNA to selectively silence pathological genes within primary endothelial cells without inducing systemic cytotoxicity. Created with Figurelabs.

Beyond synthetic nanocarriers, genetically engineered plant‐derived exosomes are emerging as a highly biocompatible alternative due to their minimal immunogenicity and excellent tissue penetration (Schwarz et al. 2025). The pioneering development of V‐Onex—constructed by engineering a VCAM‐1‐binding peptide (VHPK) onto the surface of onion‐derived extracellular vesicles (Onex)—demonstrates excellent homing specificity, selectively accumulating within atherosclerotic endothelial layers to suppress baseline inflammatory markers (Choi and Rhee 2025) (Figure 4A). This plant‐derived platform holds significant translational potential for the encapsulation and targeted delivery of endothelial‐specific TLR4 small‐molecule inhibitors or siRNAs. Furthermore, pairing nanotechnology with stimulus‐responsive release mechanisms offers a sophisticated tool for clearing senescent vascular tissue. Aptamer‐drug conjugates engineered to respond exclusively to elevated lysosomal β‐galactosidase activity ensure that senomorphic or senolytic payloads are unlocked solely inside senescent endothelial cells, preventing off‐target toxicities in adjacent healthy tissue (Munoz‐Espin et al. 2018). Functionalizing mesoporous silica nanoparticles or polymeric nanocapsules with localized stimulus‐responsive linkers allows for controlled, lesion‐specific drug release at sites of mechanical or metabolic cardiovascular injury, correcting localized angiogenesis deficiencies and restoring long‐term microvascular perfusion (Li et al. 2024; Pham et al. 2021) (Figure 4B).

**FIGURE 4:**
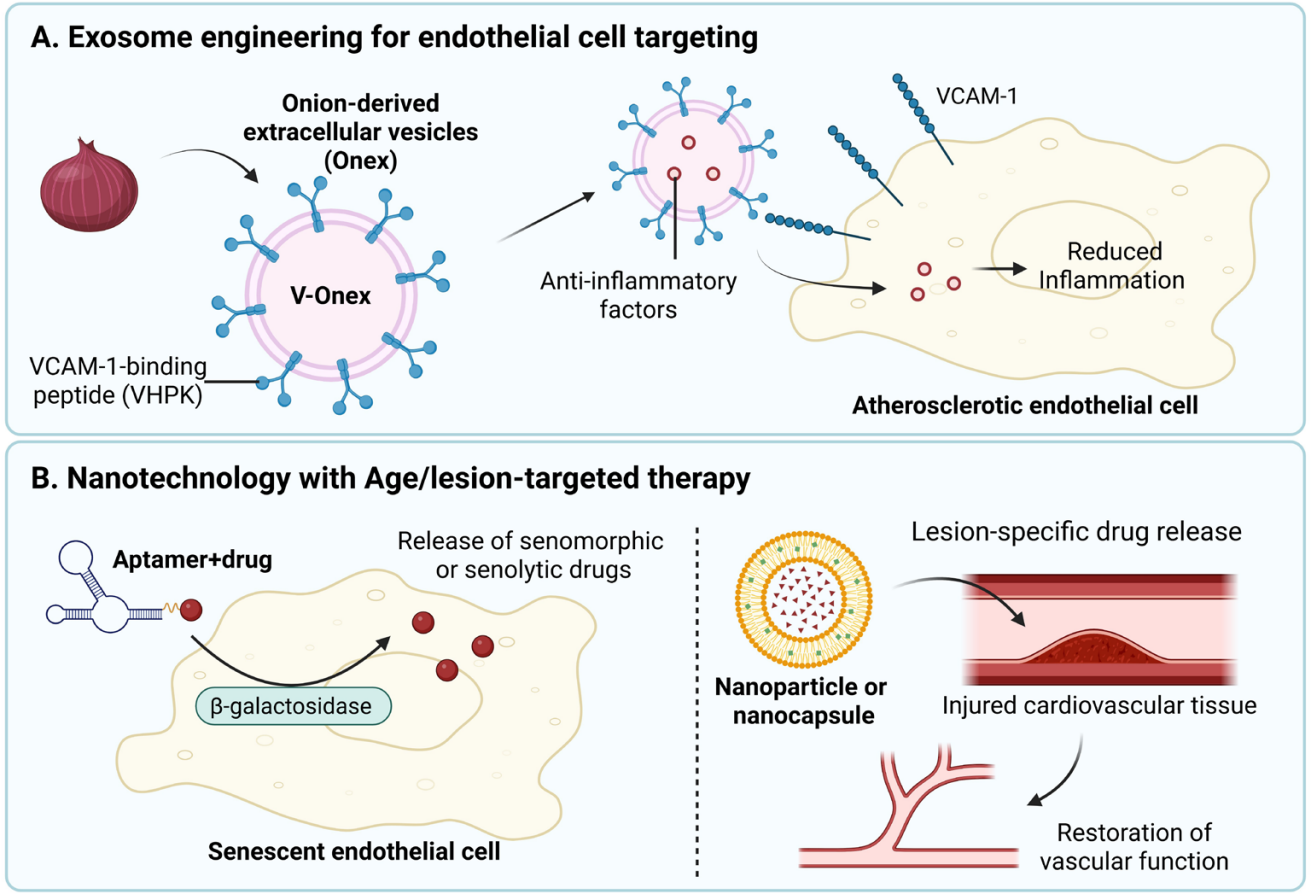
Advanced biomimetic exosome engineering and stimulus‐responsive senotherapeutic platforms. Targeted senotherapeutic platforms designed to localize therapeutic action within damaged vascular niches. (Top) V‐Onex engineering, featuring VCAM‐1‐binding peptides (VHPK) conjugated onto onion‐derived extracellular vesicles (Onex) to selectively target atherosclerotic lesions for the delivery of anti‐inflammatory cargoes. (Bottom Left) Aptamer‐based senolytics utilizing aptamer‐drug conjugates that are selectively cleaved and activated by elevated lysosomal β‐galactosidase within senescent endothelial cells, enabling targeted elimination of senescent populations. (Bottom Right) Stimulus‐responsive nano drug delivery systems (NDDS) designed for controlled payload release triggered by specific microenvironmental stimuli at the site of cardiovascular injury, restoring endothelial perfusion. Created with http://biorender.com/.

### 6.2 Limitation of Targeted Delivery Therapy

These findings highlight the translational promise of targeted exosome‐ and nanoparticle‐based therapies to selectively bind senescent ECs and silence TLR4. However, critical limitations remain to be overcome for clinical application. Although exosomes offer high bioavailability and the capability to load a diverse range of therapeutic cargoes, they suffer from challenges in mass production and purification, as well as the risk of rapid in vivo clearance before reaching the target site (Meng et al. 2020; Serrano et al. 2025). In contrast, nanoparticles allow for versatile chemical synthesis, enabling precise structural tuning and surface modification for optimal loading efficiency, alongside advantages in quality control and long‐term storage. Nevertheless, nanoparticles face constraints regarding mass scalability and the size of deliverable cargoes, while tracking their in vivo distribution and toxicity remains a challenge. To circumvent these drawbacks, hybrid technologies fusing both platforms have been proposed (Lim et al. 2014; Zamboni et al. 2004). Through further optimization steps—such as precise targeting of senescent ECs, selective loading of TLR4 inhibitors, and rigorous in vivo toxicity profiling—this integrated approach is expected to be effectively applied to endothelial senescence‐induced diseases.

## 7 Conclusion

The paradigm of TLR4 has evolved from a simple host‐defense immune sensor into a highly dynamic, context‐dependent rheostat governing endothelial homeostasis and chronological aging. Evidence across pulmonary, retinal, dermal, and cardiovascular systems demonstrates that vascular aging is driven not by the mere presence of TLR4 signaling, but by its localized hyperactivation and dysregulated ligand‐receptor kinetics. While systemic pan‐TLR4 inhibition creates fatal immunodeficiencies and metabolic disruptions, precision medicine offers a rational path forward.

Leveraging the convergence of dual‐targeted nanocarriers, stimulus‐responsive linkers, and biomimetic engineered plant exosomes to deliver TLR4‐silencing cargoes exclusively to senescent ECs represents a highly viable strategy for treating age‐related micro‐ and macrovascular diseases. Realizing this clinical translation requires a deeper biochemical understanding of how endothelial TLR4 integrates epigenetic, metabolic, and transcriptional networks within distinct cellular niches. This comprehensive review establishes the conceptual blueprint for precise, next‐generation endothelial senotherapeutics, moving the field past simple receptor blockade toward targeted spatiotemporal restoration.

## Author Contributions

**Hyo‐Jin Kim:** writing – original draft and writing – review and editing. **Jeong‐Hyung Lee:** review and editing. **Cheol Hwangbo:** project administration, review and editing.

## Funding

This work was funded by the National Research Foundation (NRF) of Korea (Grant RS‐2021‐NR061371, Grant RS‐2024‐00462318) and the Research Resurgence under the Glocal University 30 Project at Gyeongsang National University in 2025.

## Conflicts of Interest

The authors declare no conflicts of interest.

## Data Availability

The authors have nothing to report.
